# Does an Unconventional Source with a High Silica Content (Obsidian) or a High Alumina Content (Bauxite) Affect the Type of Potassium Zeolite Formation? Preliminary Data on Synthetic Products’ Efficiency in Ammonium Removal

**DOI:** 10.3390/molecules31152716

**Published:** 2026-08-05

**Authors:** Claudia Belviso, Sawssen Slimani, Antonio Lettino, Maryam Abdolrahimi, Luca Peruzzo, Francesco Cavalcante, Lorenzo Maccarino, Chiara Bisio, Davide Peddis, Federica Maraschi, Michela Sturini

**Affiliations:** 1 National Research Council—CNR, 00185 Roma, Italy; 2 Università di Genova, Dipartimento di Chimica e Chimica Industriale & INSTM RU, nM2-Lab, University of Genova, 16146 Genova, Italy; 3 Istituto di Struttura della Materia—ISM-CNR, nM2-Lab Monterotondo Scalo, 00015 Roma, Italy; 4 Istituto di Geoscienze—CNR, 35127 Padova, Italy; 5 Department of Science and Technological Innovation, University of Piemonte Orientale, 15121 Alessandria, Italy; 6 CNR-SCITEC Institute of Chemical Science and Technology “Giulio Natta”, 20133 Milano, Italy; 7 Dipartimento di Chimica, Università di Pavia, 27100 Pavia, Italy

**Keywords:** obsidian, bauxite, potassium zeolites, hydrothermal process, vapour phase crystallization method, ammonium removal

## Abstract

In this paper, obsidian (OS) and bauxite (BX) are used to synthesize zeolites via KOH prefusion, followed by a conventional hydrothermal process (HY) or an unconventional vapor-phase crystallization method (VPC) at 90 °C. The results indicate that, due to their chemical and mineralogical composition, both natural sources prove to be suitable raw materials for potassium zeolite synthesis. However, the higher silica content in obsidian is decisive for the formation of merlinoite (MER topology) and, subordinately, merlinoite-chabazite intergrowths (MER-CHA), whereas the higher alumina content in bauxite controls chabazite (CHA topology) synthesis. Moreover, our hypothesis is that the lower water content in the VPC method enhances the Al concentration during the dissolution of pre-fused raw materials, thus favouring the formation of zeolite N (EDI topology) in BX, the raw material with the highest Al content. The synthetic products were further investigated for their magnetic properties and potential application in ammonium removal from polluted water. The predominant magnetic phases (i.e., ferrimagnetic or antiferromagnetic) demonstrate that the magnetic properties are mainly governed by the transformation pathways of iron-bearing phases, which are controlled by water availability during synthesis. In turn, this gives the possibility of tailoring both structural and magnetic order through careful selection of the raw material and synthesis conditions, while simultaneously enabling efficient ammonium removal from contaminated water.

## 1. Introduction

Obsidian (OS) is a volcanic glass rich in silica, whereas bauxite (BX) is a natural deposit resulting from weathering under warm and humid conditions characterized by the presence of alumina phases such as boehmite and gibbsite, together with iron phases mainly consisting of hematite and goethite. Due to their mineralogical and chemical composition, the literature has documented the use of these natural sources for zeolite synthesis [1,2,3,4,5,6], including our previous papers [7,8]. In this study, we demonstrate that the chemical composition of unconventional natural sources, particularly their silica and alumina contents, together with water availability during synthesis, governs the formation of potassium zeolites. To elucidate these effects, obsidian (OS) and bauxite (BX) were pre-fused with KOH and subsequently subjected to hydrothermal (HY) or vapor-phase crystallization (VPC) treatments at 90 °C. Zeolites are microporous tectosilicates whose structures are characterized by a SiO_4_−AlO_4_(PO_4_) tetrahedra framework with channels and/or cages usually occupied by extra-framework cations and/or water molecules. Among potassium zeolites, merlinoite, chabazite-K, and zeolite N are the most diffuse.

Merlinoite is a small-pore potassium zeolite characterized by high adsorption and ion exchange capacity. Although it can be found in nature, many studies have documented the synthesis of a phase that closely resembles the natural form: W zeolite [9,10,11]. This type of zeolite was synthesized using silica sources and aluminum precursors [12,13,14,15], as well as natural [16,17,18,19] and waste materials [20,21,22,23]. To our knowledge, merlinoite formation from obsidian has been documented only by Kawano and Tomita [24], who investigated the alteration of the volcanic glass in both NaOH and KOH solutions at 150 and 200 °C. Chabazite is a zeolite that typically contains Ca and Na as dominant extra-framework cations, although the series also include chabazite K. Natural potassium chabazite is a highly rare mineral, but this type of zeolite can be easily synthesized in laboratories [25] using different raw materials ranging from pure sources to wastes [20,26,27]. To the best of our knowledge, this is the first report of chabazite synthesis from obsidian and bauxite precursors. Edingtonite is a natural fibrous zeolite of the EDI type [28,29]. Zeolite N, also known as Linde Type F or Barrer K-F [30,31], is a synthetic potassium form of an EDI-framework-group zeolite [32]. It was synthesized in the laboratory for the first time by Barrer and Baynham [33], and the authors improved the process in their following papers [34,35,36]. More recent manuscripts described zeolite N formation from clays, including kaolinite [37,38,39,40]. Literature data have not documented zeolite N formation starting from obsidian or bauxite. To date, the synthesis of zeolite N from obsidian or bauxite has not been investigated.

Due to their structure, all three of these potassium zeolite types can be identified differently as materials for selective ion exchange applications, particularly for the exchange of ammonium ions in polluted solutions. Ammoniacal nitrogen is a major water pollutant, and its presence in high concentrations is responsible for water eutrophication [41,42,43]. Therefore, its removal is a critical issue. Among the processes proposed in the literature to address this environmental problem, ion exchange and/or adsorption with natural and synthetic zeolites have been recognized as efficient.

Although a rather large body of literature has documented the effectiveness of natural zeolite as an adsorbent for the removal of NH_3_/NH_4_^+^ from aqueous solutions [44,45,46,47], among the synthetic products, zeolite N has also been widely used [48,49,50]. More recently, Probst et al. [39] indicated that the ion exchange performance of zeolite N is related to the synthetic mineral crystal size and dispersion, and the authors determined an ammonium capacity of 210 meq/100 g and an equilibration time of ca. 10 min for this type of zeolite.

Thanks to their high cation exchange capacity (CEC), natural and synthetic chabazite can be considered excellent adsorbent materials even for ammonia-contaminated water, but little data has documented this application, as has been the case for merlinoite [51,52,53].

To fill this gap, in this study, a preliminary screening test was conducted as a proof of concept to evaluate the ammonium adsorption potential of merlinoite, chabazite and zeolite N formed from obsidian or bauxite.

Building on our previous demonstration of the intrinsic magnetic properties of obsidian and bauxite [54,55], this work extends the investigation to the magnetic properties of the derived K-zeolites, providing insight into how zeolite topology and iron-phase transformations jointly determine the magnetic response of the final materials. It is worth noting that magnetic zeolites have attracted considerable attention due to the combination of their unique structural features and magnetic functionality, which makes these minerals particularly promising for the remediation of polluted waters and subsequent removal by the application of an external magnetic field [56,57,58].

## 2. Results

### 2.1. Zeolite Synthesis

The chemical composition of OS is characterized by a high percentage of SiO_2_ (74.20%) (Figure S1a), as confirmed by the X-ray pattern showing a broad band between 17 and 30° 2θ, typical of amorphous glass material (Figure S1b). The SEM image displays the morphology of obsidian characterized by irregular particles, generally with conchoidal fracture (Figure S1c), whereas magnetic data indicates a ferromagnetic-like behaviour with a very low saturation magnetization (Ms 0.96 A m^2^ kg^−1^) [7,54] (Figure S1d).

**Figure 1:**
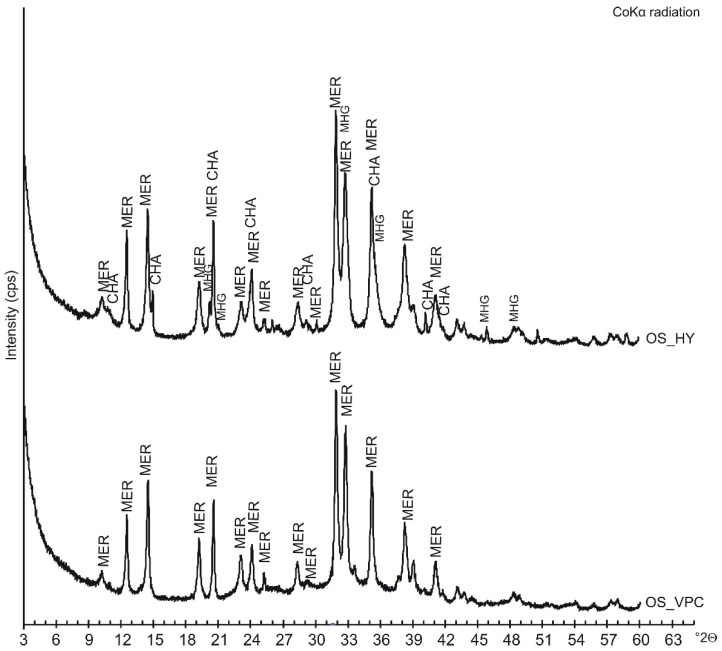
XRD patterns of OS after both HY and VPC process. MER = merlinoite; MER-CHA = merlinoite/chabazite intergrowth; MHG = maghemite.

**Figure 2:**
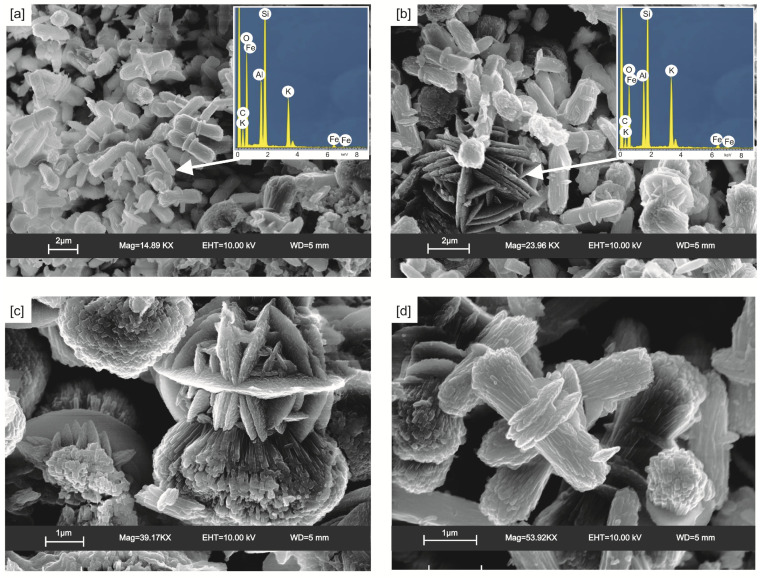
SEM images and EDX data of merlinoite [**a**]; merlinoite with isolated crystal of chabazite [**b**]; merlinoite-chabazite intergrowth [**c**,**d**] formed after OS treatment.

Thanks to its chemical and mineralogical composition (Figure S1a,b), OS is confirmed to be a very reactive raw material for the synthesis of potassium zeolite by both conventional hydrothermal process as well as by the more innovative vapour-phase crystallization method. Figure 1 in fact shows the XRD pattern of merlinoite (MER) with lower peaks attributable to chabazite (CHA) after HY treatment at 90 °C. The VPC process, instead, determines the formation of merlinoite as the main newly formed phase (Figure 1). The typical bundle-like morphology of synthetic merlinoite with the isolated star-like morphology of chabazite is shown in SEM images in Figure 2a,b. The pictures also display the presence of more or less pronounced intergrowth of MER-CHA zeolite (Figure 2c,d), particularly evident after hydrothermal treatment. The SEM images in Figure S2 confirm the almost exclusive presence of merlinoite in OS_VPC.

**Figure 3:**
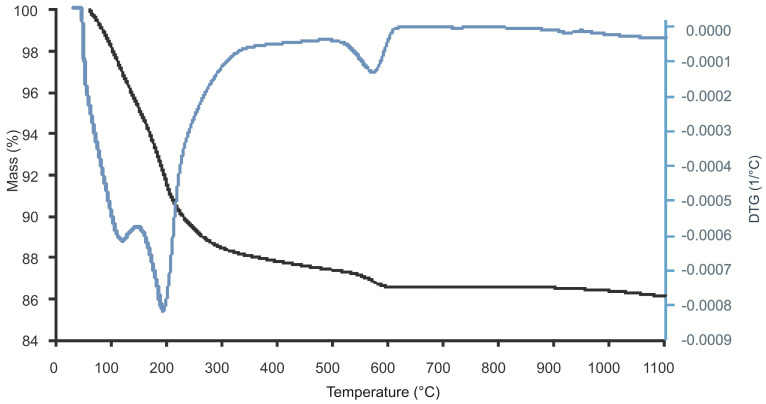
TG-DTG plots of OS_HY sample.

These results are confirmed by thermogravimetric analysis. The TG/DTG curve of OS_HY indicates an initial two-fold weight loss of up to 300 °C, with peaks at 100 and 200 °C. This should be attributed to the desorption of weakly occluded water in both MER and MER-CHA zeolites, respectively (Figure 3). The changes in slope of the TG curve at higher temperatures indicate a subsequent removal of water that occurs in overlapping and sequential steps, while the peak at around 600 °C corresponds to the loss of any residual structural H_2_O in the zeolites (Figure 3). Above 600 °C, no noteworthy thermal events are recorded on the curve, apart from a weak reaction at around 900 °C indicating the absence of further phase transitions in the zeolites and the likely presence of a small relict phase.

The chemical data of BX displays the presence of a high percentage of Al_2_O_3_ (41.41%) and Fe_2_O_3_ (15.22%) (Figure S3a), and this composition is also confirmed by an X-ray profile indicating the presence of boehmite and kaolinite together with hematite and goethite (Figure S3b) [59]. The SEM image in Figure S3c shows the nanoaggregates of bauxite whereas the magnetic characterization indicates an antiferromagnetic behaviour attributable to hematite (Figure S3d).

**Figure 4:**
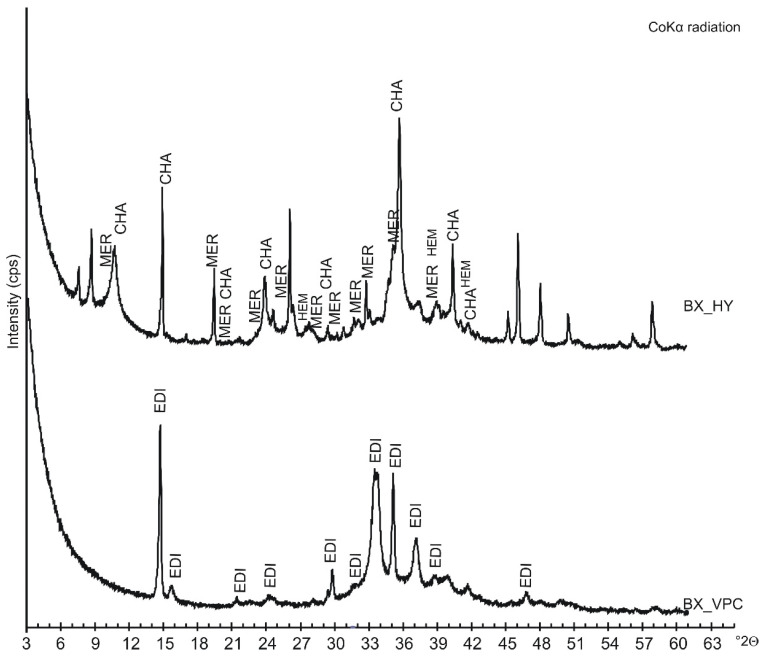
XRD patterns of BX after both HY and VPC process. MER = merlinoite; CHA = chabazite; EDI = zeolite N; HEM = hematite.

**Figure 5:**
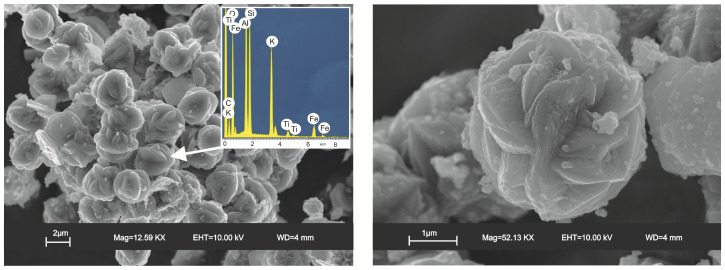
(**Left**): SEM image and EDX data of a typical chabazite obtained after hydrothermal treatment of the BX sample. (**Right**): High-magnification view showing the morphology of the same newly formed zeolite.

**Figure 6:**
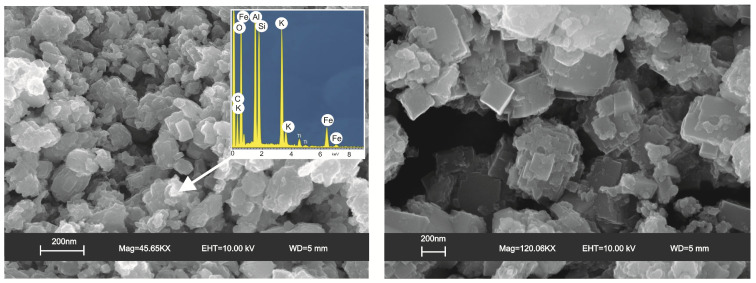
(**Left**): SEM image and EDX data of a typical zeolite N after VPC treatment of the BX sample. (**Right**): High-magnification view showing the morphology of the same newly formed zeolite.

Mineralogical results after both HY and VPC treatments show the presence of synthetic potassium zeolites. In detail, the XRD pattern after the HY process in Figure 4 displays the presence of peaks attributable to chabazite, whereas VPC methods determined the formation of zeolite N (EDI type zeolite). These results are also confirmed by the SEM images in Figure 5 showing well crystallized phase with a star-like form after hydrothermal treatment. The pictures in Figure 6 show, instead, aggregates of cubic newly formed zeolite characterizing the sample after the vapor-phase crystallization process.

**Figure 7:**
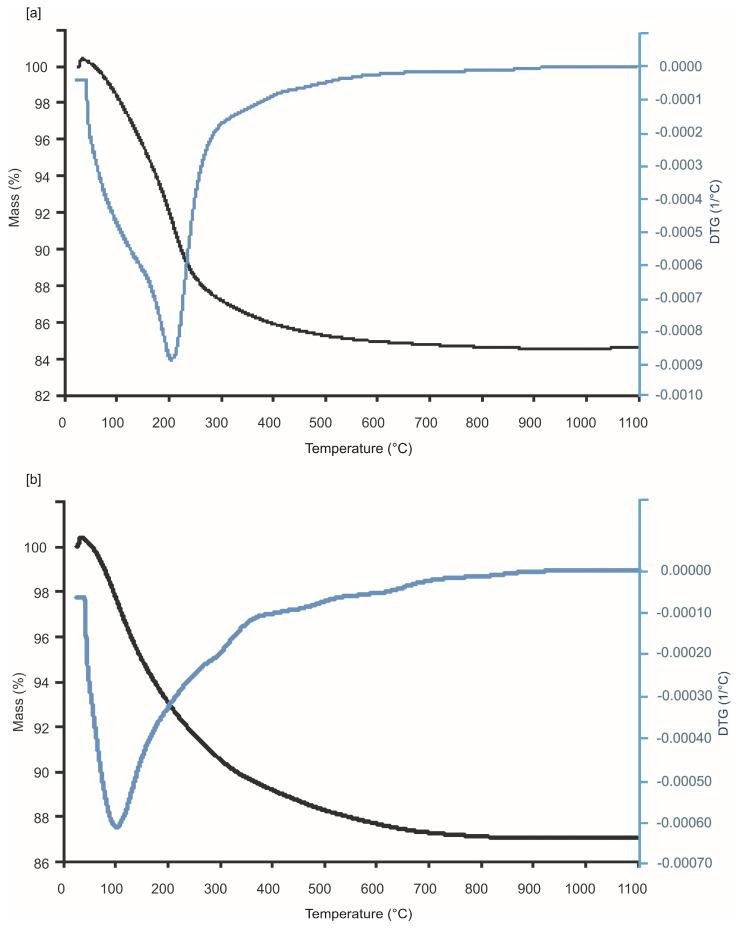
TG-DTG plots of: [**a**] BX_HY and [**b**] BX_VPC samples.

The TG/DTG curve of bauxite after the hydrothermal process displays an initial evident weight loss at about 200 °C attributable to the desorption of weakly occluded water in CHA zeolite (Figure 7a). The poorly defined fold weight loss up to 150 °C is probably due to the presence of a low amount of MER and MER-CHA intergrowth together with other clay phases also detectable on the X-ray diffraction pattern (Figure 4). The thermal analysis of the BX_VPC sample (Figure 7b) shows a different behavior, displaying TG/DTG curves with an initial evident weight loss at about 100 °C attributable to the desorption of weakly occluded water in EDI, and subsequent weak peaks that are more clear at 300 °C, from 400 to 500 °C, and at 600 °C, corresponding to the loss of any residual structural H_2_O in EDI-type zeolite and other residual products.

**Figure 8:**
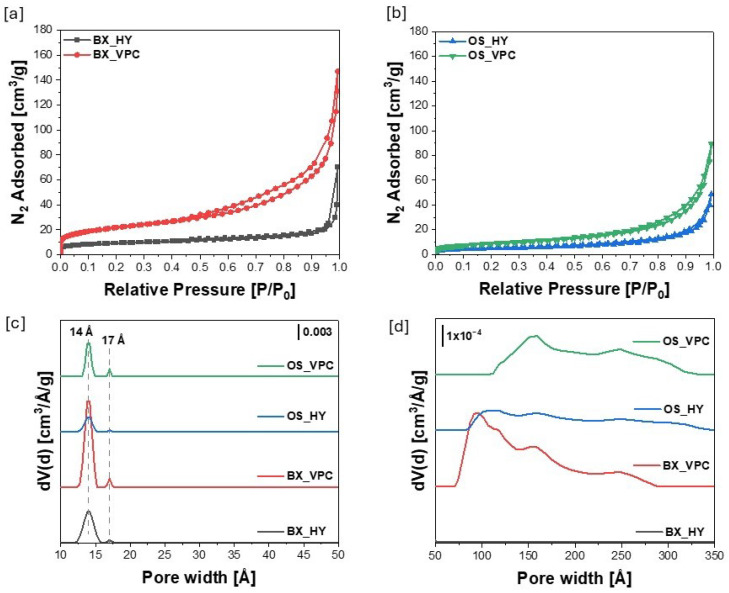
N_2_ physisorption isotherms of [**a**] BX_HY (black) and BX_VPC (red) and [**b**] OS_HY (blue) and OS_VPC (green). Pore size distributions of the four samples—BX_HY (black), BX_VPC (red), OS_HY (blue) and OS_VPC (green)—in the 10–50 Å range, evidencing microporosity [**c**] and in 50–350 Å, evidencing mesoporosity [**d**].

The textural characterisation (Figure 8a,b) of the four samples reveals a consistently low to very low porosity (Figure 8c,d), with specific surface areas (SSA) ranging from 18 to 77 m^2^/g (Table 1). The N_2_ physisorption isotherms display predominantly Type I profiles, suggesting the presence of microporous features, as also supported by the high BET C constants (R^2^ > 0.9999). However, it should be noted that the quantitative interpretation of the pore size distributions obtained by DFT analysis is inherently limited for zeolitic materials synthesized from natural aluminosilicate precursors. Their intrinsic structural and mineralogical heterogeneity, together with the relatively low nitrogen uptake and extremely small micropore volumes, reduces the reliability of pore size distributions derived from N_2_ adsorption. Nevertheless, these measurements still provide valuable qualitative insights into the relative accessibility of the porous structures and confirm the predominantly microporous character of the synthesized zeolitic materials. Therefore, the textural properties are considered as factors that may influence the accessibility of exchangeable sites and, consequently, ammonium uptake, but they cannot be regarded as the sole parameter controlling adsorption performance.

### 2.2. Magnetic Properties

**Figure 9:**
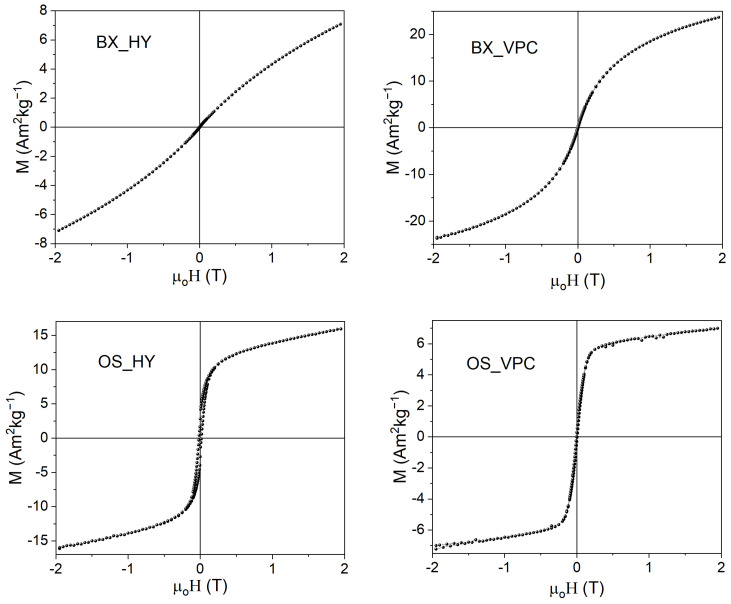
Room-temperature field dependence of magnetization of both BX and OS samples after HY and VPC treatments.

The identification of iron oxide phases in natural materials is inherently challenging because their magnetic response is intrinsically complex, arising from the coexistence of multiple mineralogical phases, broad particle size distributions, variable oxidation states, and overlapping magnetic contributions. Consequently, magnetic measurements alone cannot provide an unambiguous identification of the magnetic phases, and their interpretation generally requires the combined use of complementary characterization techniques. To this end, the magnetic properties of the synthetic products were investigated by measuring the field-dependent magnetization at 300 K (Figure 9). The identification of the iron oxide phases is inferred from the combined interpretation of the XRD results (Figure 1 and Figure 4) and magnetic measurements, supported by previous investigation of analogous materials reported in Refs. [54,59,60]. It should be noted that the iron oxide phases responsible for the magnetic behavior were not directly identified by independent techniques such as Mössbauer spectroscopy or TEM. However, the characteristic features of the M(H) curves (the presence/absence of coercivity, the S-shape vs. linear behavior, and the saturation magnetization) are consistent with the magnetic fingerprints of hematite (antiferromagnetic) and maghemite/magnetite (ferrimagnetic), as extensively documented in the literature [54,59,60]. Furthermore, the XRD peaks in the BX_HY sample (Figure 4) indicate the presence of hematite, while the difficulty in identifying distinct Fe-oxide peaks in the obsidian-derived samples is consistent with the presence of nanoscale (<5 nm) or poorly crystalline ferrimagnetic phases. Furthermore, the negligible coercivity suggests that a significant fraction of these particles may be in the superparamagnetic size regime. Based on these considerations, clear differences emerge between samples derived from obsidian and bauxite, as well as between the two synthesis methods. The OS_HY sample exhibits an S-shaped hysteresis with a low coercivity of ~165 Oe. This behaviour suggests that the magnetic response is dominated by ferro(i)magnetic oxides like maghemite (γ-Fe_2_O_3_) or magnetite (Fe_3_O_4_) inherited from the raw obsidian (Figure S1d), which has been previously attributed to nanoscale magnetite or iron-rich clusters within the volcanic glass matrix [54]. During the hydrothermal process, the combination of high alkalinity resulting from KOH prefusion, elevated water content, and a silica-rich environment facilitates the dissolution and reprecipitation of iron species. These conditions favour the neo-formation of magnetite (Fe_3_O_4_) or other ferrimagnetic iron oxides exhibiting finite coercivity. The higher water availability in the HY method compared to the VPC route appears to be a key factor, as water molecules promote ion mobility and crystal growth of iron oxides, whereas the vapor-phase method limits iron recrystallization, resulting in zero coercivity in OS_VPC sample.

On the other hand, both BX_HY and BX_VPC samples exhibit zero, while magnetization increases markedly in BX_HY, reaching ∼24 Am^2^/kg at 2 T, with respect to the BX_VPC sample (∼7 Am^2^/kg at 2 T). Moreover, the shape of the magnetization curves differs significantly: BX_HY displays an antiferromagnetic contribution, whereas BX_VPC exhibits an S-shape curve tending toward saturation at high fields, typical of ferro-ferrimagnetic behaviour. The zero coercivity observed in the BX_HY sample is directly inherited from the raw bauxite, which exhibits antiferromagnetic behaviour due to the presence of hematite, as demonstrated in our previous study [59]. In the conventional hydrothermal process, the abundant water promotes the dissolution of iron phases, while hematite (α-Fe_2_O_3_) remains thermodynamically stable under alkaline conditions, keeping the antiferromagnetic behaviour of the parent Bauxite in BX_HY sample. In contrast, the vapor-phase crystallization process operates under limited water availability, where only steam interacts with the pre-fused material. Under these conditions, incomplete iron dissolution may occur, inducing a magnetic phase transformation from α-Fe_2_O_3_ to γ-Fe_2_O_3_ (maghemite) at the surface of the hematite particles. One possible explanation is the formation of sufficiently small particles whose magnetic behavior is dominated by thermal fluctuations at room temperature, resulting in a superparamagnetic regime [61]. This induces the ferro-ferrimagnetic contribution responsible for the S-shaped magnetization curve observed in BX_VPC, whereas the linear, non-saturating behaviour of BX_HY reflects the dominance of the antiferromagnetic phase.

These findings demonstrate that the magnetic properties of synthetic zeolites formed from unconventional natural sources are governed by the transformation pathways of iron-bearing phases, which are controlled by water availability during synthesis, as well as by the original iron mineralogy of the raw material.

### 2.3. Synthetic Products’ Performance for Ammonium Removal: A Proof of Concept

The ammonium removal performance of the zeolitic materials was evaluated under environmentally relevant conditions using tap water spiked with 0.8 mg L^−1^ ammonium. Tap water was selected for its representative freshwater composition, pH, and inherent buffering capacity (see Table S1).

**Figure 10:**
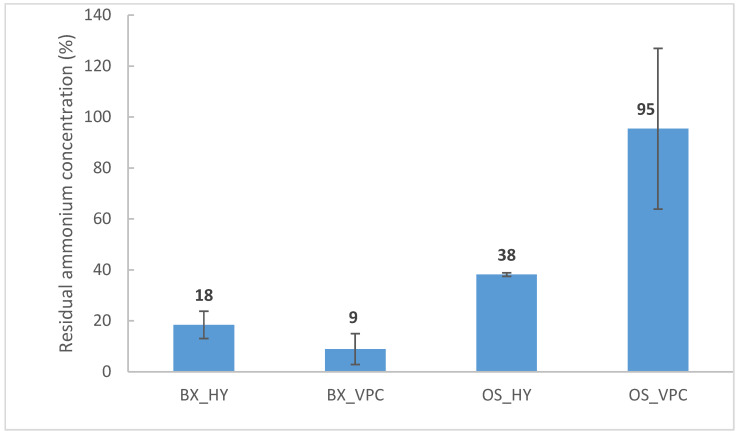
Residual percentage of ammonium after adsorption on BX_HY, BX_VPC, OS_HY, OS_VPC (Experimental conditions: 10–15 mg zeolite, 10 mL tap water sample spiked with ammonium 0.8 mg L^−1^, *n* = 3).

As shown in Figure 10, the ammonium removal efficiency of bauxite-based zeolites was markedly higher than that of their obsidian-based counterparts. Specifically, the HY treatment enhanced the performance of the obsidian-derived materials. Conversely, for the bauxite-based samples, the VPC treatment produced a more efficient zeolitic framework for ammonium removal. Moreover, in the presence of matrix macro-constituents, only BX_HY, BX_VPC, and OS_HY reduced the ammonium content, with the latter two showing the most pronounced decreases.

To ensure the reliability of these results, method blanks consisting of zeolite suspensions in unspiked tap water were prepared and analyzed. For BX_HY, BX_VPC, and OS_HY, the ammonium concentration in the method blanks remained below 0.1 mg L^−1^, with no significant difference from the matrix blank (tap water) (*n* = 3, *p* > 0.05). This confirms that neither the tap water nor these materials released significant ammonium into the system. Conversely, a significant ammonium concentration of 0.6 mg L^−1^ was detected in the OS_VPC method blank. After spiking, the analyzed OS_VPC sample exhibited a final ammonium concentration that exceeded the initially added 0.8 mg L^−1^. This unexpected trend strongly suggests a net release of background ammonium from the zeolitic framework, which cannot be readily explained at this stage. Therefore, further investigations will be conducted to fully clarify the behaviour of this material.

Regarding the system pH, it remained remarkably stable across all experiments; the mean pH of the method blanks was 8.17(7), which was very close to that of the ammonium-spiked samples (8.06(6)). Within this pH range (around 8.1)—well below the pK_a_ of the NH^4+^/NH_3_ system—the ammonium ion remains by far the dominant species (over 93%) [Standard method 4030 A APAT IRSA-CNR 2003 [https://www.irsa.cnr.it/wp/?page_id=5435] (accessed on 13 April 2026). This confirms that any loss of volatile ammonia was negligible and that the observed changes in ammonium concentration are driven exclusively by material–solution interactions.

## 3. Discussion

The results indicate that the type of potassium zeolite formed from unconventional natural raw materials such as obsidian or bauxite, in addition to the KOH concentration, is controlled by the dissolution of silica or alumina, which can be influenced by the water content in the system.

In agreement with the literature data [62], our results confirm that a more alkaline and silica-rich environment determines CHA crystallization as well as the fast transformation of chabazite into merlinoite, while in a system characterized by a lower alkalinity and high alumina concentration, the formation of CHA and EDI-type zeolite occurs, respectively. Thus, merlinoite represents the main newly formed zeolite after obsidian processing (Figure 1 and Figure 2), while chabazite and, even more so, zeolite N crystallize using bauxite as a raw material (Figure 4, Figure 5 and Figure 6). The data further indicate that water content appears to play a role in directing the type of zeolite formation. Indeed, although the VPC process at higher temperatures, such as 90 °C, is considered an intermediate mechanism between solid-state transformation and hydrothermal synthesis [63] and can even be approximated to a true hydrothermal process [8,55,64], the results indicate that steam causes less silica dissolution in our unconventional raw materials and is therefore responsible for creating a more Al-rich solution [62]. This promotes the formation of MER-type zeolite in OS_VPC (Figure 1, Figure 2a,b and Figure 3) and EDI-type zeolite in BX_VPC (Figure 4, Figure 6 and Figure 7b). The binary growth of the CHA-MER phase in the same sample (Figure 2c,d) is likely due to differences in the local SiO_2_/Al_2_O_3_ ratio concentration and alkalinity, which favored the development of the CHA or MER structure in the intergrowth crystals [62]. This behavior characterizes the OS sample mainly when subjected to the hydrothermal process, thus confirming a more diffuse presence of Si-rich growth solution with this raw material. However, the regulated interzeolitic growth and transformation is not yet fully understood and requires further investigation.

As already demonstrated in our previous papers [54,59], by exploiting the chemical and mineralogical composition of unconventional raw materials, it is possible to form zeolites with unique magnetic properties. The results, in fact, confirm that the interplay between raw material chemistry, water content, and synthesis method governs not only zeolite crystallization but also the resulting magnetic behavior.

For the bauxite-derived samples, the presence of hematite in BX_HY is supported by the characteristic XRD reflections, together with the absence of additional detectable diffraction peaks attributable to other dominant crystalline iron oxide phases. Within the detection limits of XRD, these observations indicate that hematite is the predominant crystalline phase. This interpretation is further supported by the magnetic measurements (Figure 9), where BX_HY exhibits a linear, non-saturating magnetization curve with negligible coercivity, consistent with the antiferromagnetic behavior of hematite. In contrast, OS_HY displays an S-shaped hysteresis loop with a coercivity of approximately 165 Oe, characteristic of ferrimagnetic magnetite/maghemite nanoparticles. Likewise, BX_VPC exhibits a more antiferromagnetic-like response, with a relatively high saturation magnetization, which is consistent with the presence of very small maghemite (γ-Fe_2_O_3_) nanoparticles. Such behavior is in agreement with the transformation of hematite into maghemite under water-limited or reducing conditions, as previously reported [65]. The distinct magnetic responses observed for BX_HY and BX_VPC suggest that the experimental synthesis route, particularly the amount of water used during crystallization, influences not only the zeolite phase formed but also the evolution of the associated iron oxide phases. For obsidian-derived samples, ferrimagnetism remains the dominant magnetic behavior regardless of the synthesis route. In particular, the coercivity (165 Oe in OS_HY) indicates that iron-rich magnetic phases originally present in the volcanic glass remain largely preserved under the mild alkaline synthesis conditions. Overall, these findings demonstrate that the magnetic properties of zeolites synthesized from unconventional raw materials are closely related to the synthesis pathway, particularly the water content during crystallization, providing an effective strategy for tailoring both the structural and functional properties of the resulting materials.

On the other hand, typically, natural zeolites show Type I shapes for the nitrogen physisorption isotherm that reflect microporosity and hysteresis loops indicating capillary condensation in mesopores. The presence of mesopores, particularly in both OS_HY and OS_VPC samples and the BX_VPC sample, stems from non-zeolitic impurities and voids rather than pores. However, it can be noticed that there is an increase in total porosity passing from the hydrothermal to the VPC synthetical method for two types of samples. These features, along with the constrained N_2_ uptake, align with natural zeolites: unlike their synthetic counterparts, their micropore networks are restricted by mineral phases and cations [66,67,68]. Nevertheless, it should be noted that the interpretation of DFT pore size distributions (Figure 8c,d) should be approached with a degree of caution. The implementation of these models on isotherms exhibiting low nitrogen uptake (total volume adsorbed <0.05 cm^3^/g) has been demonstrated to result in a substantial enhancement of the signal-to-noise ratio. The presence of peaks in the micropore region (such as those at 14 Å and 17 Å) is documented in the literature as a potential result of mathematical artefacts or the smooth surface assumption, which fails to account for the energetic heterogeneity of natural samples, leading to an uncertainty in the quantification of the pore network [69,70,71,72].

However, the BET data, specific surface areas, and porosity of the synthetic products (Figure 8 and Table 1) are in accordance with the preliminary experimental results on ammonium removal (Figure 10). These results show that the EDI-type zeolite, which characterizes the mineralogical composition of the BX_VPC sample, is the most performant zeolite. The observed differences in ammonium removal among the synthetic products is interpreted as the result of the combined effects of zeolite framework composition, phase assemblage, and the accessibility of ion-exchange sites.

Moreover, although it is at an early stage of investigation, experimental evidence suggests that the VPC approach offers encouraging performance compared with the standard HY process.

## 4. Experimental

### 4.1. Material and Zeolite Synthesis

A sample of rhyolitic obsidian (OS) collected at Punta delle Rocche Rosse, Lipari, Aeolian Island (Italy) [7], and a sample of bauxite (BX) collected at Murge Plateau (southern Italy) were used for the synthesis of K zeolite. In detail, OS and BX (in separate experiments) were mixed with KOH (1:1.2 ratio by weight), ground, and then heated at 600 °C for 1 h. After this step, the solid mass was crushed in an agate mortar and incubated by the hydrothermal process (HY) at 90 °C as described in our previous papers [55,59]. For the vapour-phase crystallization treatment (VPC), a portion of crushed KOH pre-fused material was placed in a ceramic raised holder inside a water bath in such a way so that the sample was contacted only with vapour from the distilled water poured into the bottom of a water bath to produce vapour molecules at 90 °C for 4 days [9].

### 4.2. Ammonium Removal Tests

Exploratory tests were conducted using a real water matrix consisting of tap water enriched with a low-level concentration of ammonium (0.8 mg L^−1^ from an ammonium chloride solution 2.5 mg L^−1^). Briefly, 10–15 mg of zeolite, exactly weighed, were suspended in 10 mL of spiked tap water and equilibrated on a roller shaker at room temperature for 24 h. After reaching equilibrium, the supernatant was separated by centrifugation, filtered through a 0.45 µm nylon filter, and analyzed using a UVmini-1240 UV-Vis Spectrophotometer (Shimadzu Corporation, Milano, Italy) according to the standard method 4030 A APAT IRSA-CNR 2003 [https://www.irsa.cnr.it/wp/?page_id=5435] URL (accessed on 13 April 2026).

In parallel, matrix blanks (tap water) and method blanks (zeolite suspensions in non-spiked tap water) were prepared and analyzed using the same procedure to determine their background ammonium content. All experiments were performed in triplicate.

### 4.3. Characterization

Preliminary information on the mineralogy of the raw materials was obtained by X-ray diffraction using a Rigaku Rint 2200 powder diffractometer (Rigaku Corporation, Akishima, Tokyo, Japan) equipped with Cu-Kα radiation. Mineralogical characterization of the synthetic products was performed using a Philips X’Pert Pro diffractometer (PANalytical B.V., Almelo, The Netherlands) with Co-Kα radiation and X’Celerator detector. The patterns were collected from 3 to 64° 2θ angles using a spinner sample holder. Morphological observations on all the synthetic products were performed using a scanning electron microscope (SEM, Zeiss Supra 40, Oberkochen, Germany) equipped with an energy dispersive spectrometer (EDS). The samples were carbon-sputtered (10 nm thick) to avoid charging of the surface. Thermogravimetric Analysis (TGA) and simultaneous Measure Heat Flow (DSC) curves were determined using a Mettler-Toledo apparatus (TG/DSC 3+). The analyses were performed from 25 °C to 1100 °C, with a heating rate of 20 °C min^−1^ under constant and controlled nitrogen flow conditions (flow rate 80 mL min^−1^). Approximately 40 mg of each sample was utilized for the analyses, with alumina (Al_2_O_3_) acting as the control and standard reference.

The chemical composition for major chemical constituents of both raw materials was determined by X-ray fluorescence, as already indicated in our previous papers [7,59], whereas nitrogen sorption measurements were performed at 77 K in the pressure range between 1 × 10^−5^ and 1 of relative pressure (P/P_0_) using a 3Flex (Micromeritics Instrument Corp., Atlanta, GA, USA) instrument. Before the adsorption phase, the samples were outgassed and thermally treated at 300 °C for 6 h. The specific surface area (SSA) of the samples was determined by the Brunauer–Emmett–Teller (BET) multipoint method considering a P/P_0_ range from 0.035 to 0.100 resulting in a R^2^ > 0.9999. The pore size distributions for all the samples were obtained by applying a DFT method on the adsorption branch of the isotherms based on an oxide surfaces with cylindrical pores model with a regularization of 0.316. The calculations were made using the software Flex—Ver. 6.03 (Micromeritics Instrument Corp.). The mesopore volume (V_meso_) was calculated as the difference between the total pore volume (V_tot_) and the micropore volume (V_micro_).

Finally, magnetic measurements were carried out by measuring field-dependent magnetization curves at 300 K by using a vibrating sample magnetometer (VSM Model 10—Microsense, Lowell, MA, USA). The sample powders were fixed by epoxy resin to prevent any movement during the measurement. All magnetization data were normalized to the mass of the magnetic fraction.

## 5. Conclusions Remarks

The results of our study confirm the effectiveness of utilizing unconventional, inexpensive, and readily available natural sources—such as obsidian and bauxite—as raw materials for the synthesis of potassium zeolites (CHA, MER, CHA-MER intergrowth, and EDI-type). Our findings suggest that the limited water availability inherent in the Vapor Phase Crystallization (VPC) method lowers silica dissolution within the raw materials. Our hypothesis is that this creates a more Al-rich environment, which favors the synthesis of merlinoite when using obsidian (OS) and EDI-type zeolites when using bauxite (BX). Furthermore, the low-temperature hydrothermal (HY) process (90 °C) facilitates the transformation of CHA into MER, promotes CHA-MER intergrowth in the silica-rich sample (OS), and regulates CHA formation in the alumina-rich source (BX). The decisive role of alkali concentration is significant in both processes, albeit in different ways. The results also demonstrate that water plays a key role in directing the synthesis process, influencing not only the crystallization pathway and phase composition but also the magnetic response of the final synthetic products. This finding highlights the importance of water content as a critical processing parameter for tailoring the functional properties of potassium zeolites. Importantly, their intrinsic magnetic behavior enables the efficient separation and recovery of all synthetic potassium zeolites from ammonium-decontaminated water by applying an external magnetic field, thereby enhancing their practical applicability and reuse potential in water remediation processes.

## Figures and Tables

**Table 1:** SSA, C constant and R^2^ values obtained from BET model and volumes of micropores (V_micro_, up to 20 Å) and mesopores (V_meso_, from 20 to 350 Å) and total pore volume (V_tot_, up to 350 Å) of the four samples.

Sample Name	BX_HY	BX_VPC	OS_HY	OS_VPC
SSA BET [m^2^/g]	34	77	18	31
C constant	213	153	194	113
R^2^ BET	0.99998	0.99994	0.999993	0.99993
V_micro_ [cm^3^/g]	0.01220	0.02133	0.00352	0.00590
V_meso_ [cm^3^/g]	0.00003	0.02749	0.01308	0.01957
V_tot_ [cm^3^/g]	0.01223	0.04882	0.01660	0.02547

## Data Availability

The data presented in this study are available on request from the corresponding author.

## Supplementary Materials

The following supporting information can be downloaded at: https://www.mdpi.com/article/10.3390/molecules31152716/s1, Figure S1: [a] Chemical composition; [b] X-ray pattern; [c] SEM image and [d] field dependence of magnetization of obsidian (OS) sample at room temperature; Figure S2: SEM images of merlinoite formed in OS_VCP sample; Figure S3: [a] Chemical composition; [b] X-ray pattern; [c] SEM image and [d] field dependence of magnetization of bauxite (BX) sample at room temperature. Knl = kaolinite; Bhm = boehmite; Gt = goethite; Ant = anatase; Hem = hematite; Table S1: Tap water pH, conductivity and composition.
