# Supplementary material for: Does an Unconventional Source with a High Silica Content (Obsidian) or a High Alumina Content (Bauxite) Affect the Type of Potassium Zeolite Formation? Preliminary Data on Synthetic Products’ Efficiency in Ammonium Removal
Source: Molecules. 2026 Aug 5;31(15):2716. doi: 10.3390/molecules31152716 (PMC13467873; doi:10.3390/molecules31152716)
Supplement: Supplementary file 1 [file molecules-31-02716-s001.zip › molecules-4426648-supplementary.pdf]

Supplementary Information

[a]

Chemical composition:Major costituents (wt.%)	
Oxides	***Wt.%***
Na_2O	4.31
MgO	0.15
Al_2O_3	13.20
SiO_2	74.20
P_2O_5	0.02
K_2O	4.94
CaO	0.96
TiO_2	0.09
MnO	0.08
Fe_2O_3	1.76
SiO_2/Al_2O_3	5.62

[b]

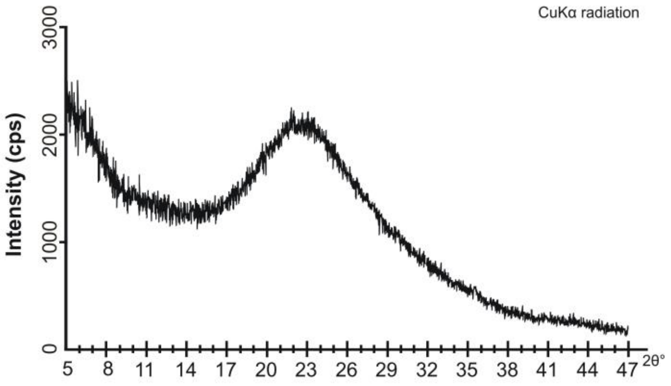

[c]

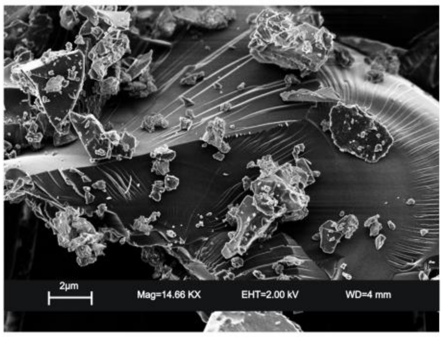

[d]

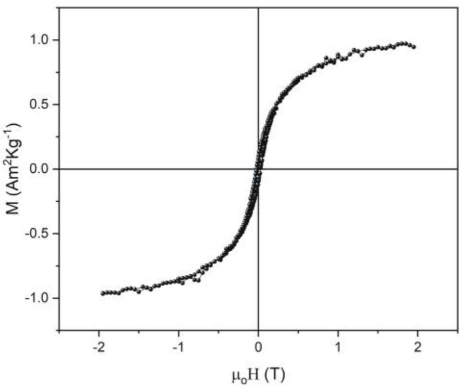

Figure S1. [a] Chemical composition; [b] X-ray pattern; [c] SEM image and [d] field dependence of magnetization of obsidian (OS) sample at room temperature.

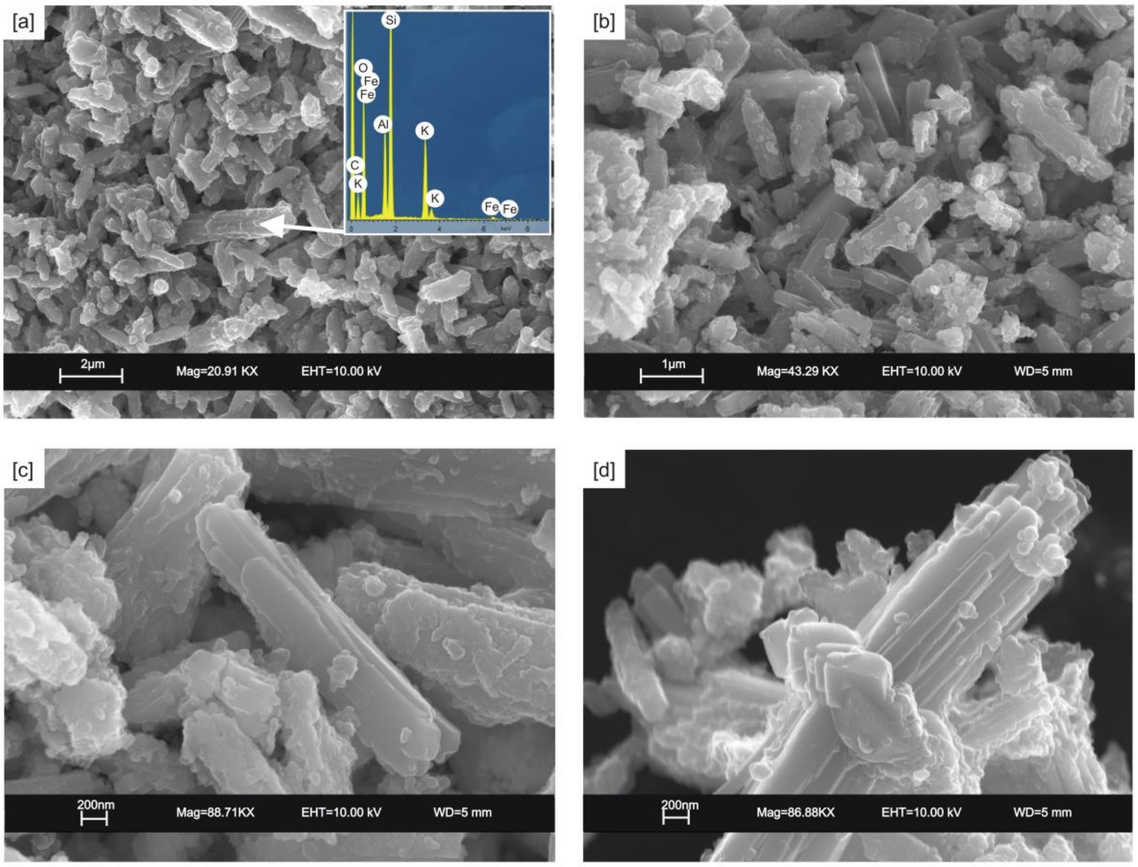

Figure S2. SEM images of merlinoite formed in OS_VCP sample.

[a]

Chemical composition:Major costituents (wt.%)	
Oxides	***Wt.%***
Na_2O	0.05
MgO	0.18
Al_2O_3	41.41
SiO_2	25.95
P_2O_5	0.05
K_2O	0.14
CaO	0.49
TiO_2	3.71
MnO	0.03
Fe_2O_3	15.37
SiO_2/Al_2O_3	5.62

[b]

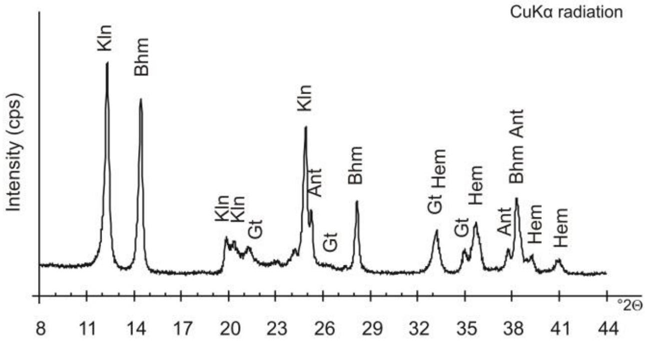

[c]

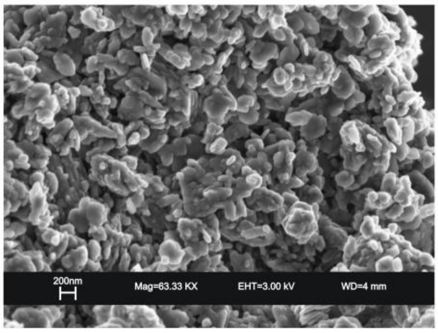

[d]

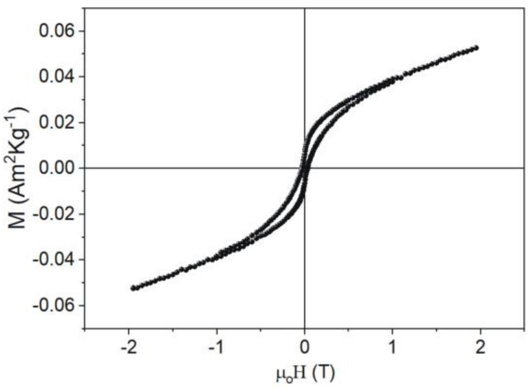

Figure S3. [a] Chemical composition; [b] X-ray pattern; [c] SEM image and [d] field dependence of magnetization of bauxite (BX) sample at room temperature.Knl=kaolinite; Bhm=boehmite; Gt=goethite; Ant=anatase; Hem=hematite

Table S1. Tap water pH, conductivity and composition.

Parameters/Ions	Unit	Tap water
pH		7.7
Conductivity at 20 °C	µS cm^{-1}	278
HCO_3^-	mg L^{-1}	195
Cl^-	mg L^{-1}	4.5
NO_3^-	mg L^{-1}	0.6
SO_4^{2-}	mg L^{-1}	5.0
Ca^{2+}	mg L^{-1}	38
Mg^{2+}	mg L^{-1}	12
Na^+	mg L^{-1}	11
K^+	mg L^{-1}	1.8
NH_4^+	mg L^{-1}	≤ 0.1
